# Craniofacial morphology and dental maturity in children with reduced somatic growth of different aetiology and the effect of growth hormone treatment

**DOI:** 10.1186/s40510-017-0164-2

**Published:** 2017-04-27

**Authors:** Sotiria Davidopoulou, Athina Chatzigianni

**Affiliations:** 1 Department of Preventive Dentistry, Periodontology and Implant Biology, School of Dentistry, Faculty of Health Sciences, Aristotle University of Thessaloniki, Thessaloniki, 54124 Greece; 2 Department of Orthodontics, School of Dentistry, Faculty of Health Sciences, Aristotle University of Thessaloniki, Thessaloniki, 54124 Greece

**Keywords:** Craniofacial morphology, Dental maturity, Short stature, Children, Growth hormone treatment

## Abstract

Children with reduced somatic growth may present various endocrinal diseases, especially growth hormone deficiency (GHD), idiopathic short stature (ISS), chromosomal aberrations, or genetic disorders. In an attempt to normalize the short stature, growth hormone (GH) is administered to these children. The aim of this literature review was to collect information about the craniofacial morphology and dental maturity in these children and to present the existing knowledge on the effect of GH treatment on the above structures.

This review demonstrated that regardless of the origin of the somatic growth retardation, these children show similar craniofacial features, such as short length of the cranial base and the mandible, increased lower facial height, retropositioned mandible, and obtuse gonion angle. On the other hand, dental maturation does not demonstrate a specific pattern. Except for the above findings, muscle alterations seem to be present in individuals with short stature, who present low body muscle mass and strength, while studies on their craniofacial muscles seem to be lacking. After GH administration, the exact amount and pattern of craniofacial growth is unpredictable; however, the facial convexity decreases, mandibular length increases, and posterior facial height increases, while tooth eruption remains unaffected. Thus, it is of great importance to gain more insight into the craniofacial growth of treated and untreated children with reduced somatic growth so that the influence of GH therapy on the various craniofacial structures could be ascertained and proper orthodontic treatment could be selected.

## Background

The growth of the human organism from the zygote stage to its culmination in adult stature is a highly complex phenomenon involving a multitude of regulatory mechanisms, which control tissue differentiation, generation, and maturation [1]. Throughout childhood and adolescence, gains in height and weight are sensitive and reasonably accurate indices of the health and well-being of an individual. In general, structure or height is a more accurate basis for evaluating the overall growth process because healthy children show wide variability in weight [1].

The causes of short stature can be familial or pathologic. The pathologic causes may be postnatal malnutrition, digestive diseases, chronic infections, endocrine causes (such as GH deficiency), chromosomal abnormalities (e.g., Turner syndrome, Down syndrome-trisomy 21, Edwards syndrome-trisomy 18, Patau syndrome-trisomy 13, and trisomy 17 mosaicism) [1, 2], and genetic syndromes (e.g., Russell-Silver, Prader-Willy, Noonan, short stature homeobox containing gene deficiency/SHOX-D, Williams, Kabuki, Leri-Weill, and skeletal dysplasias) [3, 4].

Precise terminology and the ability to distinguish normal from abnormal growth have never been more important than at present because of the increasing tend to administer growth hormone (GH) to children without any signs of growth hormone deficiency (GHD), whose heights are within or below the low normal range [5, 6]. Treatment of children and adolescents with idiopathic short stature.

Apart from the body height, the mechanisms regulating craniofacial growth and development are complex interactions between genes, hormones, nutrients, and epigenetic factors that give the craniofacial bone its final morphology, while any disturbances in this mechanism may result in a deviating growth pattern. These regulatory mechanisms initiate and direct the growth mechanisms, which deal with how new bone is developed i.e., (a) the growth pattern, which concerns change in size and shape and (b) the growth rate, which decides the amount of growth over time [7].

Studies on craniofacial growth in children with reduced somatic growth of different origins have shown that several facial structures are smaller than expected [7–10]. Growth retardation does not affect all structures to the same extent, leading to an abnormal facial morphology [7, 11, 12]. Normal craniofacial growth and craniofacial characteristics of reference groups including children of different age and sex subgroups with documented craniofacial norms are available [13] and could be used to understand the impact of somatic growth deficiency on the craniofacial region. Moreover, studies on muscle and skeletal health in children and adolescent with GHD compared to healthy groups, report affected bone and muscle mass and strength, with GH replacement therapy exerting beneficial results [14]. Some evidence also underline the particular relationship between body stature and muscle strength. Predictive equations may help with assessing the neuromuscular involvement in children suffering from various disorders, particularly those affecting their stature [15].

The development of the dentition is an integral part of craniofacial growth, even though it is not closely related to other maturational processes. Dental maturation has been shown to be mildly delayed in some short-statured children of specific causes [9, 16–20]. Occlusal characteristics have been also examined in individuals with GHD, idiopathic short stature (ISS), and Russell-Silver syndrome (RSS) and compared to the means of a normal population to detect malocclusion occurrence. The RSS cohort presented statistically significant greater mean overbite as well as mandibular and maxillary crowding compared to the general population [21].

While somatic growth in children with short stature has been well documented [22], little is known about the craniofacial development and dental maturation in these children. Moreover, further investigation is needed for the exploration of the potential role of GH treatment on the craniofacial development. The aim of this critical review is to provide the pediatricians and orthodontists, the later knowledge on the morphology of the craniofacial complex, the development of the dentition, and the potential role of GH therapy in children with growth retardation of different aetiologies.

## Review

### Craniofacial characteristics of children with growth retardation

#### Craniofacial characteristics of children with isolated growth hormone deficiency

Growth hormone deficiency can be isolated (isolated growth hormone deficiency (IGHD)) or combined with other general disorders. IGHD mutations in the genes encoding GH (GH1) can either lead to classical GHD (types IA, IB, and II) or bio inactive GH syndrome [2]. The clinical features of patients with IGHD vary with the aetiology, age at onset, and severity of the disorder. Those with congenital IGHD have characteristic growth patterns, as they display significant maturational delays and reduced somatic growth. Infants with congenital IGHD usually have normal birth length and weight. They grow normally for 3 to 6 months, but linear growth rates decelerate thereafter. Their linear growth curves deviate progressively from the mean [1]. The literature pertaining to craniofacial development suggests that GHD results in an immature facial appearance. The length and depth of the face are inappropriately small for the age, with the face maintaining a child-like convexity [19].

Patients with GH insufficiency were found to show severe growth retardation in the linear facial measurements, greatest in the posterior facial height (PFH). In the male patients, all linear measurements of the cranial base were found to be retarded and significant differences in total mandibular length, lower anterior facial height (LAFH), and total anterior facial height (AFH) were found as well as a retrognathic facial type [10]. Choi et al. [8] found that before GH treatment boys with GHD had shorter anterior and posterior cranial base lengths, mandibular ramus height, and corpus length than those in reference group. In addition, they had greater A point, nasion, B point (ANB) values. Likewise, girls with GHD had shorter anterior cranial base length and mandibular ramus height.

**Fig. 1:**
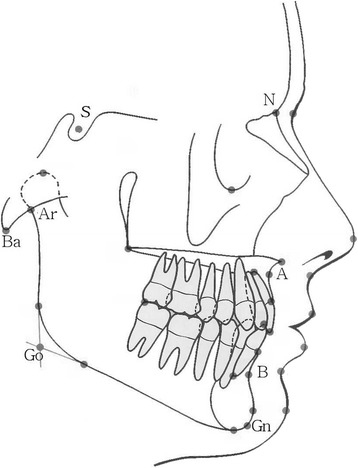
Common cephalometric landmarks used in the cephalometric analysis

**Fig. 2:**
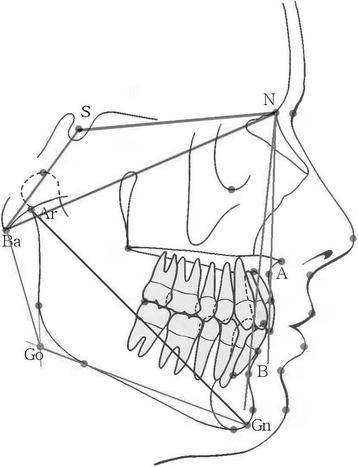
Common landmarks and reference lines used for linear and angular measurements in the lateral cephalogram

In the female patients, the anterior cranial base growth (S-N) (Figs. 1 and 2) was normal and the posterior cranial base growth was retarded [9, 23]. Pituitary insufficiency appeared to retard growth to a greater degree than it did to maturation [9]. Pirinen et al. [24] confirmed that children with GH deficiency have a smaller PFH than healthy ones. Most studies have reported relatively smaller posterior cranial bases versus anterior [18, 23]. The mandibular length is significantly reduced, primarily as a result of a smaller renal height [25]. Oliveira-Neto et al. [26] in 2011 found the total maxillary length to be the most reduced linear parameter followed by posterior cranial base length, total mandibular length, total PFH, total AFH, mandibular corpus length, and anterior cranial base length.

#### Craniofacial characteristics of children with idiopathic short stature

Short stature has a varied aetiology, and attention has been given to those children who have no recognizable disorder that may have contributed to a reduction in the statural height. The term idiopathic short stature (ISS) has been applied to those children. According to Spiegel et al. [9], the craniofacial findings of children with idiopathic short stature were similar to those of patients with GHD, but there was less retardation. Considerable facial retardation was evident, especially in the PFH measurement. Additionally, the cephalometric analyses of the short-statured boys showed that all linear measurements, except for the anterior cranial base and the mandible corpus (Go-Gn) (Figs. 1 and 2), were significantly smaller in the Kjellberg et al. [7] study group. The PFH was proportionately smaller than the AFH, and the LAFH was proportionately larger than the upper anterior facial height (UAFH). A flat medial and lateral cranial base (N-S-Ba, N-S-Ar) and a large gonion angle (Ar-Go-Gn) (Fig. 2) were significant characteristics of the short-statured boys. Both the maxilla and the mandible were significantly retropositioned, especially the mandible, resulting in facial retrognathia [7]. Especially, girls with ISS had greater ANB values than those with GHD and reference groups, indicating that girls with ISS had a more severe skeletal class II facial profile [8].

#### Craniofacial characteristics of children small for gestational age and/or with intrauterine growth retardation

A newborn is considered small for gestational age (SGA), when the measurements of its weight or length or both are at least two standard deviations (−2SDs) below the mean for the gestational age and sex, based on data derived from a reference population [27]. SGA must be differentiated from intrauterine growth retardation (IUGR). A child who is born SGA has not necessarily suffered from IUGR and infants who are born after a period of IUGR are not necessarily SGA [28]. The prognosis for their recovery depends upon the timing, severity, and duration of the intrauterine insult.

About 80% of infants born small-for-gestational age show growth acceleration during the first 6 months of postnatal age and, as a rule, they grow normally thereafter. However, infants who may show persistence of an abnormal growth rate until the age of 2 years are likely to be short throughout childhood and adulthood [1, 29–31].

The term IUGR suggests diminished growth velocity in the fetus occurring in utero, as documented by at least two intrauterine growth assessments. IUGR can be due to abnormal embryologic development of the placenta, factors related to maternal health, intrauterine infections, congenital abnormalities of major organ systems, chromosomal defects, and a variety of growth deficiency syndromes of genetic or unknown origin [1].

Somatic growth in SGA children has been documented, but little is known about the craniofacial development in these individuals. Pioneering studies suggest that facial growth in SGA children is retarded in a way similar to that in children with hypopituitarism. Especially, PFH measurements were found to be reduced [4]. Van Erum et al. [32] evaluated the craniofacial growth in short SGA persons. All linear measurements, especially the mandible and the craniofacial base, were decreased, except for the LAFH. The SNB angle was also found decreased indicating a retropositioned mandible. The cephalometric findings explain the clinical impression that SGA children demonstrate a typical facial pattern: small dimensions in the lateral aspect within a divergent face. In this study, no relationship was found between the age of the subjects and the craniofacial deficits, indicating that this condition has a prenatal or early developmental origin [32].

#### Craniofacial characteristics of short-statured children of genetic origin

The most common genetic disorders, which cause growth retardation and short stature and with available studies in the literature focusing on their craniofacial characteristics, are presented below.

#### Silver-Russell syndrome

Children with Silver-Rusell or Russell-Silver syndrome (SRS or﻿ RSS) demonstrate typical physical features: low birth weight and/or length for gestational age, characteristic triangular face, relatively prominent forehead, small mandible, clinodactyly of the fifth finger, and a variable body asymmetry [33]. These children grow consistently close to or below the 3rd centile without demonstrable endocrine abnormality. Cephalometric analysis in SRS children demonstrated small linear dimensions, which were most pronounced in PFH and mandibular length, resulting in a clockwise rotation and retroposition of the mandible [34].

#### Turner syndrome

Turner syndrome is a relatively common disorder that occurs in 1:25,000 female births and is caused by complete or partial absence of the X chromosome. There are also rare cases with structurally abnormal X chromosomes [35]. In addition to the short stature, which is the main characteristic, cranial growth reduction has been registered [36]. Women with Turner’s syndrome exhibit a flattened cranial base, bimaxillary retrognathism, and a posteriorly inclined mandible [37]. Unlike AFH, PFH was significantly decreased altering the usual PFH/AFH ratio [38]. It is uncertain whether reduced growth capacity of facial height caused by an X chromosome deficiency has any influence on the direction of mandibular growth rotation or whether an underdeveloped PFH represents just a consequence of backward growth changes in the mandible. Bimaxillary retrognathism as well as a skeletal class I jaw relationship are also described [38].

Women with Turner syndrome show a significantly shorter posterior cranial base (S-Ba), a normal anterior cranial base, and a significantly larger cranial base angle (N-S-Ba) (Figs. 1 and 2). The head circumference was normal. The maxilla was retrognathic, posteriorly rotated, and positioned closer to the sella. UAFH was normal, but the upper posterior face height (UPFH) was significantly shorter. The length of the mandible was significantly shorter (Go-Gn and Gn-Ar). The mandibular angle was normal [39]. Laine and Alvesalo [40] have reported that the alveolar arch of the mandible is broader and shorter in relation to the maxilla, where the most predominant finding is a narrow arch.

#### Familial dwarfism

In 1966, Laron et al. [41] described a syndrome of familial dwarfism which was indistinguishable both clinically and in many of the laboratory findings from pituitary dwarfism but in which there were abnormally high plasma concentrations of immunoreactive human growth hormone (IR-HGH). One of the typical features of this syndrome is the small face and mandible, which gives the false impression of a large head. Some of their findings are in contrast to the typical findings of patients with achondroplasia where normal anterior cranial base length, recessed maxilla, and a prognathic mandible that was anteriorly displaced, but of normal size with a normal gonial angle, were found [42]. Since then, several other syndromes of familial dwarfism have been described [43], but their clinical characteristics may vary and further investigation is needed.

### Craniofacial characteristics of children with growth retardation after the therapeutic administration of growth hormone

The stimulation of growth by injection of human growth hormone in child with hypopituitarism was first reported 45 years ago. The imminent approval of biosynthetic GH by the Food and Drug Administration portents a revolution in GH therapy [44], as it has increased the available supplies of biosynthetic growth hormone. As a consequence, some short children, who do not meet the classic criteria of GH deficiency, are now being selected for GH substitution therapy [6].

Treatment of GH deficiency produces a “catch up” phenomenon in both height and skeletal maturation, especially during the first year of replacement therapy [45]. Although their response to therapy is not as pronounced, short normal children also show a positive response to GH regardless of their diagnostic differences [46–50].

Studies of craniofacial measurements in treated IGHD children are limited. The available evidence suggests that facial convexity decreases, mandibular length increases, and LFH increases leading to normalization of the profile and the facial appearance, while the arch width remains constant and the cranial base length shows minimal change [18, 19]. Pool et al. [18], on the contrary to other investigations, noted that maxillary length was of normal size before GH treatment and that it increased disproportionately with treatment. Cantu et al. [25] evaluated the differential growth and maturation of craniofacial structures in IGHD children during treatment with replacement therapy. Height, skeletal age, AFH, PFH, and posterior cranial base length demonstrated significant differences between the untreated and treated groups. The patient’s age at the start of GH replacement therapy had a significant effect for 7 of the 11 measurements. There was no effect of starting age on anterior and posterior cranial base lengths, AFH, or maxillary length (ANS-PNS). PFH showed a greater improvement than either AFH or posterior cranial base length. In their study, antero-posterior growth of the maxilla was not affected by GH therapy as previously suggested [18, 19]. Their results imply that facial dimensions with the greatest growth potential display the greatest catch-up responses in IGHD patients treated with replacement. Moreover, therapy should commence as early as possible before the development of detrimental discrepancies [25].

According to de Faria et al. [47], GH treatment with standard doses in GH-deficient patients can improve the facial profile in retrognathic patients and does not lead to facial disharmony although extremity growth, mainly involving the feet, can occur.

Finally, catch-up growth following GH therapy appears to be most pronounced for tissues under intrinsic control. Other craniofacial structures under alternate control show varying responses to therapy, some of which may potentially result in undesirable, non-physiological craniofacial growth patterns [25].

In ISS children, most measurements improved toward norm after GH treatment [8]. However, the amount of mandibular growth was significantly greater in girls with ISS than in those with GHD during GH treatment. As a result, the AFH in girls with ISS was greater even than those in reference group after 2 years of treatment, resulting in an undesirable long face [8].

Regarding SGA children, it has been shown that postnatal catch-up growth occurs in the majority of those patients [30]. Several of these studies have provided evidence that the catch-up growth occurs mainly during the first two years of life and that further catch-up is limited. Approximately 10–20% of SGA children develop into short adults. It is this group that could benefit from a treatment strategy aimed at increasing height. GH treatment in short SGA children leads to craniofacial catch-up growth, which is particularly pronounced in regions where interstitial cartilage is involved. The result is that the facial profile is improved as it becomes less convex [11, 32].

In syndromic short-statured children and especially in Turner syndrome, the effect of the therapeutic use of GH in the development of the craniofacial complex has not been studied in depth. It seems that mandibular growth may be more affected by GH treatment than is maxillary growth, and thus, it should be carefully monitored over long-term GH therapy [51]. The anterior and posterior cranial base length was found not to have changed significantly during treatment. The maxillary length remained almost the same. The length of the mandible increased significantly during treatment. This must have been mainly due to increased vertical growth since the horizontal dimension did not increase. The initially posteriorly rotated mandible showed an anterior rotation, although the normal position was not reached. No indications were found for an increase in the disproportionate growth or for excessive chin growth as a sign of acromegaly during GH treatment [39]. Nevertheless, the results of Juloski et al. [52] proved that, although positive, the effects of GH therapy do not overcome the craniofacial characteristics related to Turner syndrome.

### Dental maturity and eruption in short-statured children and the effect of growth hormone treatment

Studies on tooth eruption and dental maturity indicate that both may be related to GH secretion [25, 32, 53]. The curve of the daily rhythm of tooth eruption [49] was similar to the spontaneous pattern of GH secretion [53]. However, the rate of tooth formation does not appear to increase during GH treatment [54]. Whether GH deficiency directly influences dental development and tooth eruption is still under debate.

As indicated, dental development of children with GHD is characteristically less affected than either somatic growth or skeletal maturation [16, 18]. The mean delay in dental age was less than 1 year in the study of Cantu et al. [25]. These results are similar to the findings of other studies [9, 16, 18, 19]. Furthermore, there was no significant GH treatment effect on dental maturation [43], as shown in prior studies [18–20]. The smaller delay and the lack of subsequent therapeutic response would indicate that dental age is less influenced by and less sensitive to GH than somatic and craniofacial growth. The consistency of results across studies demonstrate that the dental age findings along with those relating to skeletal age and height of this limited sample of children are representative of the affected population as a whole [25].

Regarding children with ISS, most studies indicate that they demonstrate a slightly retarded dental age, but the degree of the retardation varies and there is no clear relationship between dental, bone, and chronological age [9, 55, 56]. Additionally, GH therapy in ISS children did not have a significant influence on tooth formation although it had a significant influence on acceleration or gain in stature [56]. Kjellberg et al. [7] noted that dental maturity and tooth eruption were delayed 1.3 years with no significant differences between the ISS and the GHD deficient children.

Finally, for non-GHD children who were born small for gestational age Van Erum et al. [11, 32] found normal scores for dental maturity. Children with Russell -Silver syndrome have a dental eruption delay. The first extensive dental examination of Turner patients was performed by Filipsson and Hall [57], who reported a tendency for early eruption of teeth. This has been verified by later studies [58, 59], in some of which dental development and eruption of patients with Turner syndrome was found to be significantly advanced by 0.63 years relative to control subjects [43]. Mitbo and Halse [60] found that the timing of tooth eruption was normal in young girls with Turner syndrome (<10 years), but delayed after 10 years of age. The timing coincides with the decreases in GH secretion after the age of nine due to lack of normal increase of GH during puberty in these girls indicating that the GH secretion influences the timing of tooth eruption.

## Conclusions

In conclusion, children with short stature of different origins develop similar craniofacial characteristics. The craniofacial characteristics include smaller lengths of the cranial base, the mandible, and proportionately smaller posterior than anterior facial height, retrognathic face, and posterior rotation of the mandible. Results on the length of the maxilla were contradictory. The dentoalveolar anomalies involved slight retarded dental maturity and eruption, tooth crowding, anterior open-bite tendency, and high incidence of distal bite. Craniofacial muscles may also be affected but evidence is still premature. After GH administration, the exact amount and pattern of growth is unpredictable; however, the facial convexity decreases, mandibular length increases, and posterior facial height increases, while tooth eruption remains unaffected.

Generally, there are not many studies available in the literature analyzing the craniofacial growth, craniofacial muscle alteration, malocclusion type, and dental maturity of short-statured children and the influence of the GH treatment on the above structures. Future studies should be planned, focusing especially on the overall above characteristics. According to our knowledge, it seems to be also a lack of data available regarding craniofacial muscle pathology in individuals with short stature. A recent study of Gutroneo et al. (2012) [61] revealed the expression of muscle-specific integrins in masseter muscle fibers during malocclusion disease. Thus, considering the important function of masseter muscles, these evidence could be useful for future studies. Records should be collected before and after GH administration, according to the specific treatment protocol of each short-statured patient group, and compared to a non-affected control group with normal skeletal and dental features, to understand the complex mechanisms regulating patient’s phenotype, function, and response to GH treatment.

It is of great importance to all specialists and also orthodontists dealing with short-statured children to be aware of the clinical findings on the craniofacial region and to gain more insight into the craniofacial growth and tooth formation in children with reduced somatic growth. Moreover, proper knowledge of the influence of GH therapy on the craniofacial structures is essential so that proper timing of orthodontic treatment with proper orthodontic appliances could be selected.

## Abbreviations

AFH: Anterior facial height
GH: Growth hormone
GHD: Growth hormone deficiency
IGHD: Isolated growth hormone deficiency
IR-GH: Immunoreactive growth hormone
ISS: Idiopathic short stature
IUGR: Intrauterine growth retardation
LAFH: Lower anterior facial height
LFH: Lower facial height
LPFH: Lower posterior facial height
PFH: Posterior facial height
SGA: Small for gestational age
SRS: Silver-Russell syndrome
UAFH: Upper anterior facial height
UPFH: Upper posterior facial height
